# Mendelian randomization analysis and validation supports MEGF9 and MLLT11 as potential targets for the treatment of varicocele and male infertility

**DOI:** 10.3389/fendo.2024.1416384

**Published:** 2024-09-26

**Authors:** Bin Cai, Dalin Sun, Weimin Deng, Yihan Jin, Hongle Zhao, Dong Xing, Yuanyuan Liu, Baofang Jin

**Affiliations:** ^1^ Andrology Department of Integrative Medicine, Zhongda Hospital, School of Medicine, Southeast University, Nanjing, Jiangsu, China; ^2^ Reproductive Medicine Center, Zhongda Hospital, School of Medicine, Southeast University, Nanjing, Jiangsu, China; ^3^ Department of Andrology, Shaanxi Provincial Hospital of Traditional Chinese Medicine, Xi’an, Shaanxi, China; ^4^ Medical College of Southeast University, Nanjing, Jiangsu, China

**Keywords:** varicocele, male infertility, genetic causal effect, Mendelian randomization analysis, Bu Shen Huo Xue Prescription

## Abstract

**Objective:**

A growing body of research suggests a link between varicocele and male infertility (MI). However, current evidence is mainly based on retrospective studies, which are prone to interference from confounding factors and cannot establish causal relationships. Mendelian randomization (MR) studies on the causal relationship between varicocele and MI are very limited. Therefore, this study conducted a two-sample MR study to elucidate the causal effect between the two.

**Methods:**

Download the data set GSE216907 from the GEO database, and use R software to screen differential genes in normal and varicocele tissue samples. The drug targets of Bu Shen Huo Xue Prescription (BSHXP) were derived from the Herb database. All genetic datasets were obtained using publicly available summary statistics based on individuals of European ancestry from the IEU GWAS database. MR analysis was performed using MR Egger, weighted median (WM) and inverse variance weighted (IVW) methods to assess the causal relationship between exposure and outcome and to validate the findings by comprehensively evaluating the effects of pleiotropic effects and outliers. The renal vein constriction method was used to establish a pathological model of varicocele infertility. The drug was administered continuously for 60 days and the relevant indicators of the rats were observed.

**Results:**

Obtain two therapeutic targets for varicocele through intersection analysis: MEGF9 and MLLT11, and were verified by molecular docking. MR analysis showed that MEGF9 was positively associated with MI (MR Egger, OR: 1.639, 95% CI: 1.124-2.391, *P* = 0.024; WM, OR: 1.235, 95% CI: 1.003-1.521, *P* = 0.047). MEGF9 is also positively associated with MI (IVW, OR: 1.35, 95% CI: 1.069-1.705, *P* = 0.012). Sensitivity analysis showed no heterogeneity and horizontal pleiotropy. The expression of MEGF9 and MLLT11 increased in the varicocele model group, while the expression decreased after treatment with low, medium, and high doses of BSHXP. In addition, the sperm number, motility, morphology, and fertility of rats in the model group were significantly lower than those in the control group (*P*<0.05). After BSHXP treatment, all indicators were significantly better than those of the model group (*P*<0.05).

**Conclusion:**

In conclusion, this study indirectly supports that varicocele causes MI. BSHXP inhibiting MEGF9 and MLLT11 may become a potential therapeutic target for alleviating varicocele and MI.

## Introduction

Infertility has become a common global problem, affecting 10-15% of couples, with approximately 40% of cases being caused by the male factor (1). Male infertility (MI) defined as the inability to conceive after 1 year of regular unprotected periods, is the main indication for assisted reproductive technology (ART) (2). Common causes and risk factors of MI have been hypothesized and confirmed in various studies (3, 4), including aging, testicular dysfunction, lifestyle factors (such as tobacco and obesity), endocrine diseases, gonadotoxic exposure, and congenital anatomical factors (5). An insightful review suggests that MI symptoms may serve as future markers of mortality and health status (6). Although MI is recognized as a condition that has an impact on the quality of life of both partners of the infertile couple, there are fewer data on specific quantification and impact compared to other health-related conditions.

Clinically, varicocele is defined as abnormal dilatation, elongation, and tortuosity of the pampiform venous plexus within the spermatic cord. Varicocele is generally accepted to be the most common correctable cause of MI, affecting up to 20% of healthy men and 40% of men with primary infertility (7). More than half a century of research has shown that varicocele has a negative impact on sperm function (8), testicular histology (9), reproductive hormones (10) and semen quality (10). Systematic reviews and meta-analyses have shown that varicocele damages sperm DNA, thereby compromising the reproductive potential of affected men (11). Surgical treatment such as varicocele ligation is considered to be one of the most effective treatments, but it has disadvantages such as large trauma and slow recovery. Studies have also shown that varicocele is underdiagnosed in men assessed for infertility (12). Most men with varicocele are still able to have children, so it is particularly important to find effective and reliable drug treatments. According to traditional Chinese medicine theory, the occurrence of varicocele is closely related to kidney deficiency and blood stasis (13). Bu Shen Huo Xue Prescription (BSHXP) Yangjing Capsule is a traditional Chinese medicine compound medicine that aims to improve patients’ symptoms by tonifying the kidney and replenishing qi, promoting blood circulation and removing blood stasis (14). BSHXP contains a variety of Chinese herbal medicines such as Epimedium brevicornu Maxim, Rehmannia glutinosa, Hominis Placenta, Astragali Radix, and Polygonatum sibiricum. These ingredients can significantly improve the spermatogenic function of the testicles of animals with kidney yang deficiency and kidney yin deficiency, reduce testicular histological damage in rats with testicular dysfunction, and improve fertility (15). Among them, Herba Epimedii has the pharmacological effect and safety of improving kidney yang deficiency and sexual dysfunction (16). According to the Dictionary of Chinese Medicine Formulae, the Apriori algorithm was used to summarize the high-frequency Chinese medicines for the treatment of infertility, including Rehmannia glutinosa, Angelicae Sinensis Radix and other Chinese herbal medicines (17). In recent years, some studies have shown that BSHXP can alleviate testicular damage caused by varicocele through mechanisms such as regulating testicular blood flow, anti-oxidation and anti-inflammatory mechanisms (18). However, the benefits and harms of varicocele treatment for adult infertile men remain controversial (19). There are many theories about the potential pathophysiology of varicocele-induced infertility, and the exact link between the two remains unknown. Randomized controlled trials (RCTs) should be an ideal study design to confirm the causal relationship between varicocele and MI. However, conducting RCTs in reality faces difficulties. Mendelian randomization (MR), a method that uses genetic variation to measure causal exposure relationships among disease risk variables, can remove confounding bias inherent in observational studies. MR minimizes the effects of measurement error and directional causality. Since these instrumental variables (IVs) remain constant after conception and are expected to be free from potential founders, the MR approach overcomes some limitations of traditional epidemiological studies. To elucidate the causal direction between varicocele and MI, a two-sample MR study was performed using the genome-wide association study (GWAS) database in this study.

## Materials and methods

### Study design

**Figure 1:**
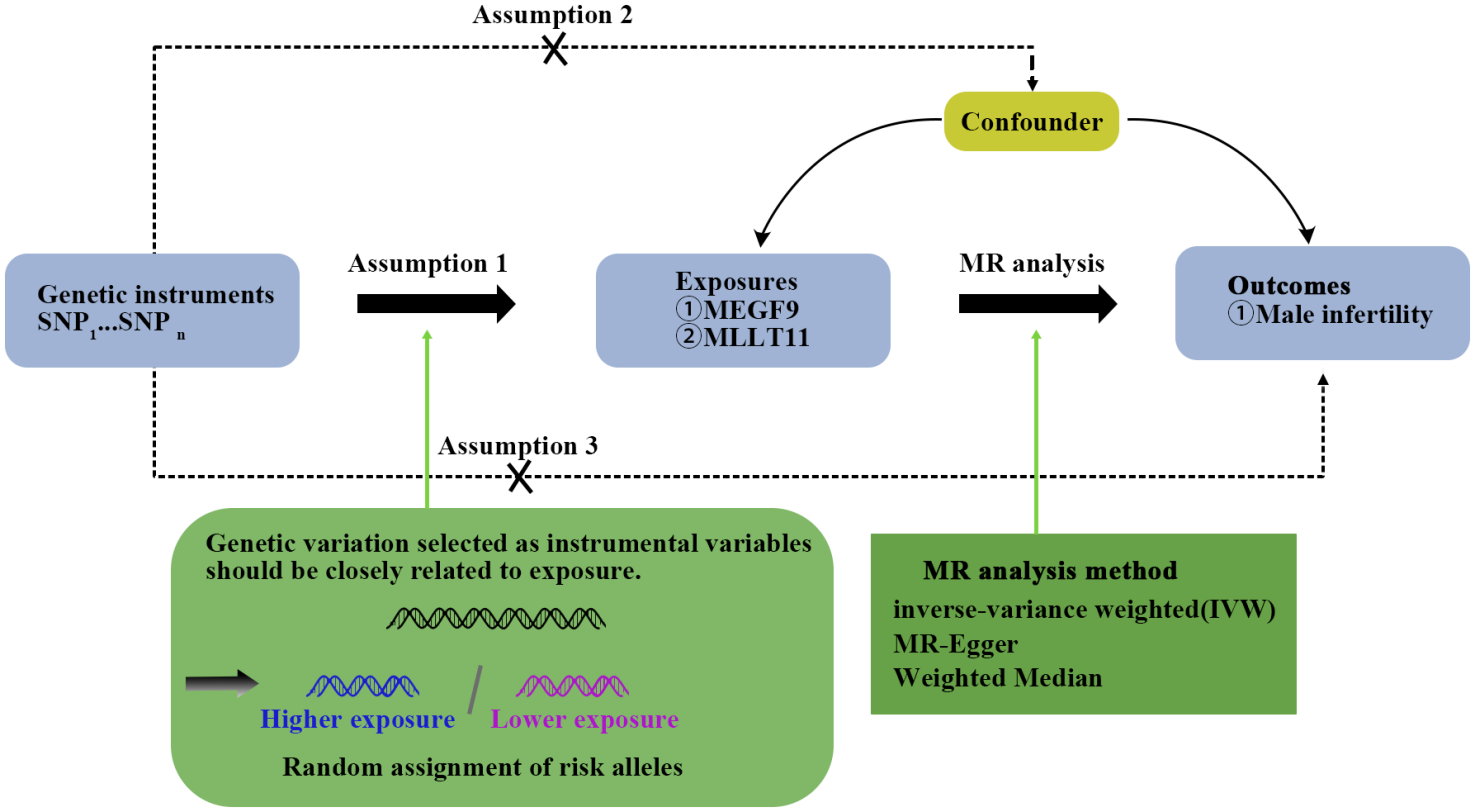
Mendelian randomization (MR) analysis design. This design assumes an association between exposure and outcome but not confounders. SNP stands for single nucleotide diversity.

Due to the lack of GWAS data on varicocele, we chose to conduct MR analysis between key genes and MI. Standard MR analysis requires that the following three model assumptions must be met (20): 1) Single nucleotide polymorphisms (SNPs) used as instrumental variables (IVs) are significantly associated with MEGF9, and MLLT11 and reach the genome-wide significance threshold; 2) SNPs were independent of confounding factors; 3) SNPs were only associated with MI through MEGF9 and MLLT11, but not through other pathways. The first hypothesis can be tested directly using observational data. However, the last two assumptions are often difficult to test in practice. In this study, we validated our findings using the MR approach under different model assumptions. **Figure 1** shows a schematic diagram of the MR study for this study.

### Data sources

Dataset GSE216907 contains gene expression data from adult testicular tissue samples from normal (N), obstructive azoospermia (OA), non-obstructive azoospermia (NOA) and varicocele (VA) donors. Information about Bushen Huoxue Recipe comes from the Herb database (http://herb.ac.cn/). The analysis in this study was based on the publicly available online database of the IEU Genome-Wide Association Studies (GWAS) (https://gwas.mrcieu.ac.uk/) (21). Data for “MEGF9”, “MLLT11” and “Male infertility” came from GWAS including 13,556,363, 16,379,540, and 10,894,596 SNPs respectively. The sample size of MEGF9 and MLLT11 has not been reported yet. Details can be found in **Table 1**.

**Table 1:** Detailed information of GWAS summary statistics used in the analysis.

Phenotypes	GWAS ID	Consortium or study	Population	Year	Sample size	Number of SNPs
ENSG00000106780 ENSG00000213190 Male infertility	eqtl-a-ENSG00000106780 eqtl-a-ENSG00000213190 finn-b-N14_MALEINFERT	NA NA Neale Lab	European European European	2018 2018 2021	— — 176,008	13,556,363 16,379,540 10,894,596

GWAS, Genome-Wide Association Studies. NA, not applicable.

### Selection of instrumental variables

GWAS have fueled the rise of MR by identifying genetic variants that can be used as instrumental variables in a two-step framework to determine whether pending DNA methylation is present along a causal pathway between exposure and disease. In our two-sample MR approach, instrumental variables (IVs) underwent a rigorous selection process:

1. We chose SNPs significantly associated with the exposure (*P* < 5×10^-8^, meeting the genome-wide threshold).
2. Independent SNPs were identified, and the PLINK clustering method was applied with a clustering threshold (r^2^ < 0.001, clump distance > 10,000 kb) to remove SNPs causing deviations due to linkage disequilibrium (LD). SNPs were further filtered based on allele frequency and incompatible alleles of the palindrome, eliminating low-quality SNPs.
3. To evaluate weak instrument bias, F statistics were calculated, and only SNPs with F < 10 were considered weak and subsequently removed (22).

These stringent steps in IV selection aim to enhance the reliability and robustness of the instrumental variables used in our MR analysis.

### MR analysis

This study mainly used MR-Egger, Weighted median (WM) and IVW (Inverse variance weighted), a total of 3 algorithms for two-sample MR analysis to evaluate the causal relationship between MEGF9, MLLT11 and Male infertility. The MR-Egger approach provides a progressively consistent measure of causal effect, adjusting for horizontal pleiotropy by pooling individual SNP-specific Wald ratios via adaptive Egger regression. The WM method produces progressively consistent causal effect estimates by using the weighted median of the Wald ratios, provided that at least 50% of the variants meet the effective IV of the exclusion limits. IVW was identified as the primary method, the weighted linear regression model, in the absence of IV In the case of horizontal pleiotropy, the results have high confidence (22).

### Sensitivity analysis

The results of the primary analysis were validated by several different MR approaches with different model assumptions:

1. Level multiplicity occurs when the genetic variation associated with the exposure of interest directly affects the outcome through multiple pathways other than the assumed exposure. Effectiveness. MR Egger’s intercept test was used to determine the level of pleiotropy among these SNPs (23);
2. Cochran’s Q statistic of the IVW method was used to assess the heterogeneity of IV causality estimates among individuals, if the Cochran’s Q test’s *P* Values < 0.05, heterogeneity was detected. The smaller heterogeneity indicates that MR estimation is more reliable;
3. Leave-one-out (LOO) analysis can be used to evaluate the influence of single SNP causal estimation, and each exposure-related SNP is sequentially discarded by LOO analysis to repeat IVW analysis.

### Renal vein constriction method to establish pathological model of varicocele

Thirty male clean-grade SD rats (3 months old), weighing 180-220g, were adaptively fed for 1 week, and the model was established after observing no abnormalities. Anesthetize by intraperitoneally injecting 40 mg/kg sodium pentobarbital (dose: 3%). The rats are fixed on a splint in a supine position and routinely disinfected. An abdominal incision is made to carefully expose the left renal vein, adrenal vein, testicular vein and inferior. For the vena cava, use microvascular forceps to carefully separate the deep surface of the vein from the inside of the superior renal vein and testicular vein and the outside of the inferior vena cava. Thread a 3/0 silk thread, place a homemade metal probe on the anterior wall of the renal vein, and then connect the vein with the metal. The probe is ligated together, the probe is pulled out, the renal vein is partially recanalized, and the abdominal wall is sutured layer by layer. In the control group, six rats only underwent separation of the left renal vein without constriction of the left renal vein. All animals were approved by the Ethics Committee of Zhongda Hospital Affiliated to Southeast University School of Medicine.

### Grouping and medication

According to the basic pathogenesis of varicocele infertility, which is kidney deficiency and blood stasis, we have formulated BSHXP (Yinyanghuo, Wangbuliuxing, Muli, Danggui, Huangqi etc.) as the basic prescription for the treatment of this disease. It nourishes the kidneys to produce body fluids and activates blood circulation to remove blood stasis, thus improving the quality of reproductive essence. Dosage and method: ① Control group (n=6): Inject physiological saline into the stomach at a rate of 1 ml/100g body weight, once a day; ② BSHXP low-dose group (n=6): Dilute BSHXP extract with double-distilled water into a liquid containing crude drug 0.6g/ml, gavage at 1 ml/100g body weight, once a day; ③ BSHXP medium dose group (n=6): dilute BSHXP extract with double distilled water into a liquid containing crude drug 1.2g/ml, gavage at 1 ml/100g body weight, 1 time a day times; ④ BSHXP high-dose group (n=6): Dilute the BSHXP extract with double-distilled water into a medicinal solution containing crude drug 2.4 g/ml, and administer it into the stomach at a rate of 1 ml/100g body weight, once a day. Each group was administered intragastric administration 48 days after the completion of modeling and continued for 60 days.

### Detection of sperm count, viability, and morphology in the epididymis of rats in each group

Quantity detection: Remove the tail of the left epididymis, cut it several times, place it in 2 ml of physiological saline, leave it for 30 minutes, and shake gently. Then add 5 ml of normal saline to dilute, and count the sperm number on a hemocytometer, expressed as ×10^6^/ml. Viability test: Make a small cut in the tail of the left epididymis near the end of the vas deferens, and a small amount of semen will flow out. Take a slide and dip it in a small amount of semen. After diluting it with physiological saline, immediately observe the sperm activity under the microscope and count the sperm. Activity percentage. Morphological observation: Take a small drop of semen on a glass slide, and pull the sample drop into a smear. Stain with 0.5% gentian violet alcohol for 3 minutes, dry naturally, wash with water and then undergo microscopic examination.

### Western blot

Group-frozen rat peripheral blood, penile tissue, and semen samples were removed from liquid nitrogen. According to the instructions of the protein extraction kit, use RIPA lysis buffer to extract the total protein of the sample. Subsequently, the above extracted protein solution was subjected to polyacrylamide gel electrophoresis (SDS-PAGE, 10% separation gel), transferred to PVDF membrane, blocked at 37oc for 30 minutes, and the primary antibody MEGF9 (1:500, 29924-1-AP), MLLT11 (1:1000, PA5-103027) and GAPDH (1:3000, AG019-1) were incubated overnight at 4ter After elution of the primary antibody, the membrane was incubated with the secondary antibody at 37ti for 90 min, and then the membrane was incubated with the horseradish peroxidase-conjugated secondary antibody at 37ti for 90 min. Finally, Odyssey two-color infrared laser imaging system and Alpha software were used to scan and conduct semi-quantitative analysis of protein bands.

### ELISA detection of TXB_2_ and 6-Keto-PGF_1_a levels

About 5 ml of blood was taken from the rat orbit, placed in a test tube with indomethacin-EDTA. Na_2_ anticoagulation, centrifuged at 3500 rpm for 15 minutes at 4tnu and the plasma was separated and stored in a freezer at -20eze Operate according to the instructions of the reagent TXB_2_ and 6-Keto-PGF_1_a detection kit.

### Statistical analysis

MR is based on the principle of random distribution of genetic variants. When the frequency of SNPs is highly consistent with the changes in exposure variables, it can be preliminarily considered that SNPs are related to exposure variables. All statistical tests were two-sided and were considered to show statistical significance at a *p*-value<0.05. The analyses were conducted using the “TwoSampleMR” package (Version: 0.5.6) and “MRPRESSO” in R software (Version: 4.1.0) “ package (Version: 1.0). The β value represents the effect size of each unit increase in the exposure variable on the outcome variable. A positive value indicates that an increase in exposure is associated with an increase in the outcome, and a negative value indicates that an increase in exposure is associated with a decrease in the outcome. The 95% confidence interval represents the uncertainty range of the effect estimate. If the confidence interval does not include zero, the effect is generally considered significant. The Cochran Q statistic in the inverse variance weighted model was used to test the heterogeneity between the specific estimates, and then the MR-Egger and Outlier methods were used to explore the horizontal pleiotropy. If the P value is greater than 0.05, it indicates that there is no significant pleiotropy and heterogeneity, which further enhances the credibility of the results. GraphPad Prism 8 software was used to analyze the differences among the groups.

## Results

### Screening potential therapeutic targets for varicocele

**Figure 2:**
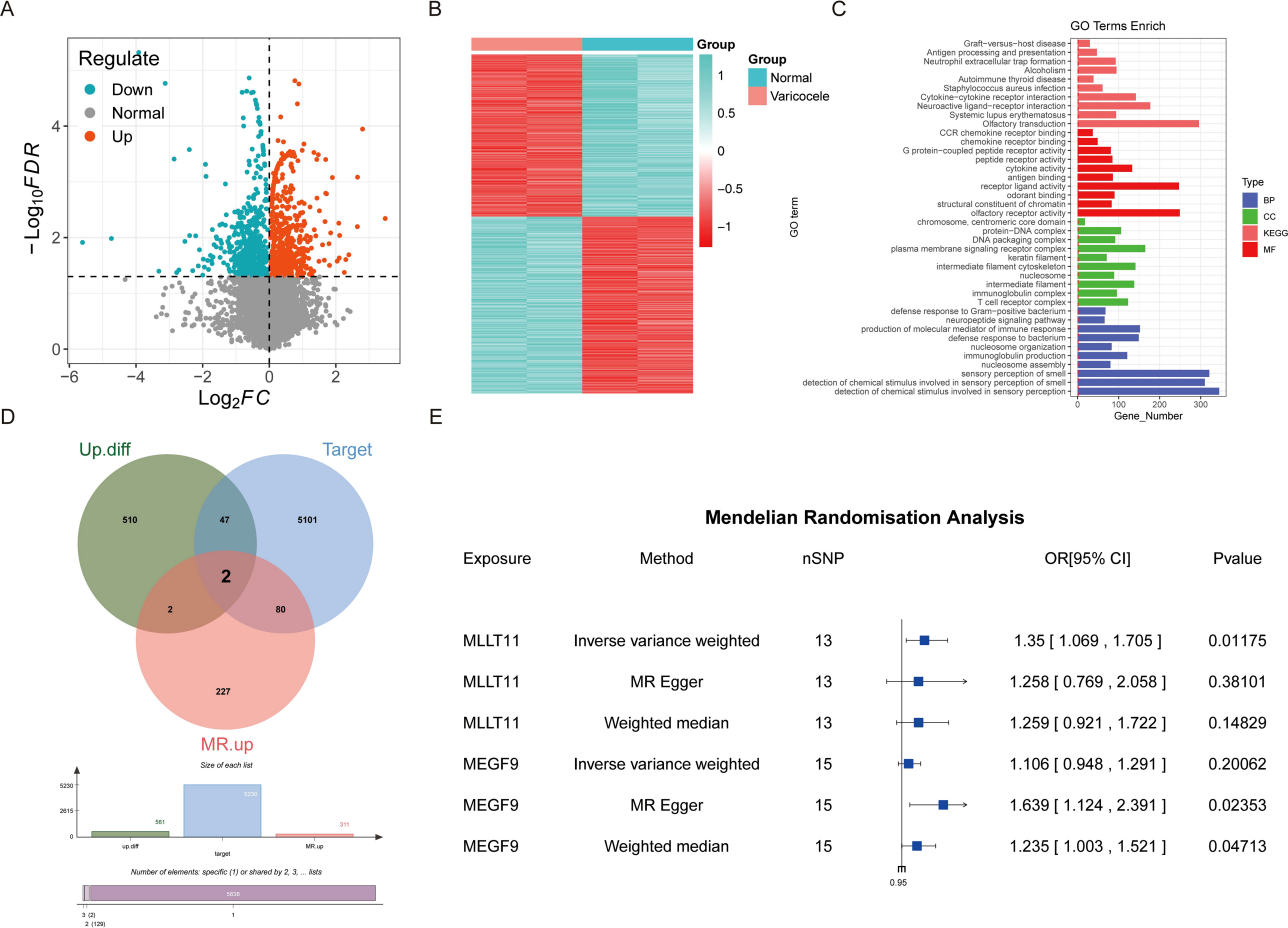
The impact of varicocele-related drug targets (Bu Shen Huo Xue Prescription) on male infertility. **(A)** Volcano plot; **(B)** Heat map; **(C)** Functional enrichment analysis of differential genes; **(D)** Intersection analysis of up-regulated genes, genes positively correlated with MI in Mendelian randomization, and herbal targets of Bushen Huoxue Recipe; **(E)** Mendelian randomization forest plot of intersection genes.

In order to study the relationship between varicocele and MI, we first screened out 1076 differential genes based on the gene expression data in the GSE216907 data set, of which 561 were up-regulated and 515 were down-regulated (**Figure 2A**). The differential genes were then displayed in heat maps and functionally annotated (**Figures 2B, C**). 311 genes in MR that were positively correlated with the occurrence of MI were retained from the 561 up-regulated genes (**Supplementary Table S1**). Based on the theory of traditional Chinese medicine and the basic pathogenesis of varicocele, a team developed a traditional Chinese medicine for nourishing the kidney and activating blood circulation- BSHXP, which is a more reasonable formula for the treatment of varicocele infertility. The full prescription consists of 11 traditional Chinese medicines including 13.3% Yinyanghuo (Herba Epimedii Brevicornus), 13.3% Wangbuliuxing (Semen Vaccariae Segetalis), 13.3% Muli (Concha Ostreae (calcined)), 10% Danggui (Radix Angelicae Sinensis), 10% Huangqi (Radix Astragali Mongolici), 6.7% Shayuanzi (Semen Astragali Complanati), 6.7% Ziheche (Placenta Hominis), 6.7% Huangjing (Rhizoma Polygonati Sibirici), 6.7% Lizhihe (Semen Litchi), 6.7% Shuizhi (Hirudo), and 6.7% Shudihuang (Radix Rehmanniae Preparata) (24, 25). Through network pharmacology analysis combined with Herb database, 5230 herbal targets of Bushen Huoxue prescription were retrieved. After intersection analysis among the three, two key genes were finally obtained: MEGF9 and MLLT11 (**Figure 2D**). The forest plot showed that MEGF9 and MLLT11 significantly promoted the occurrence of MI (**Figure 2E**). In addition, intersection analysis was also conducted on varicocele down-regulated genes, drug targets of Bushen Huoxue Recipe, and genes in MR that were negative for disease occurrence. However, the results showed that the selected intersection genes were 0 (**Supplementary Figure S1**).

### Varicocele drug targets

**Figure 3:**
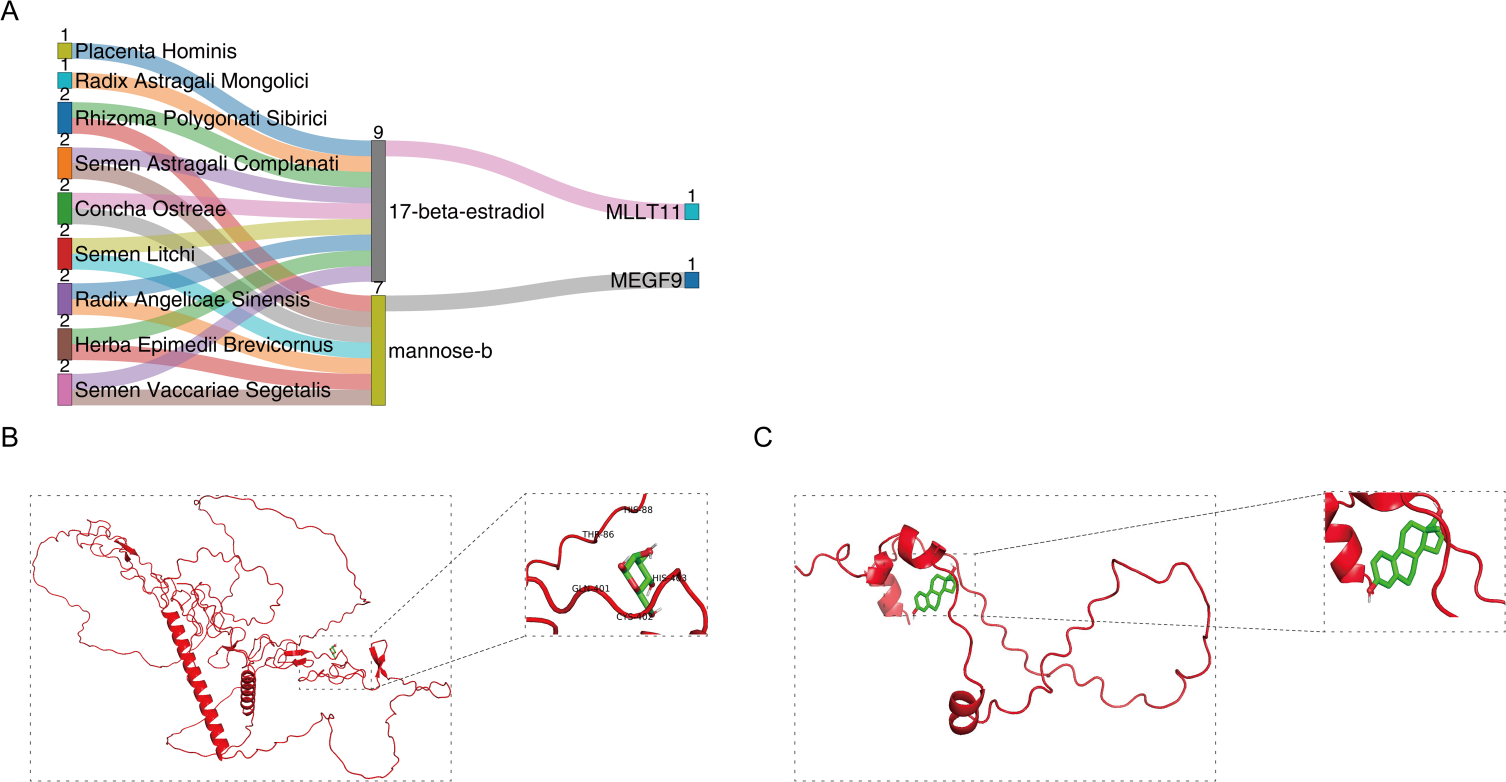
MEGF9 and MLLT11 as potential therapeutic targets for varicocele. **(A)** BSHXP-TCM monomer-target gene; **(B)** MEGF9 and mannose-b molecular docking, MEGF9-mannose-b binding energy: -1.74 kcal/mol; **(C)** MLLT11 and 17-beta-Estradiol molecular docking, MLLT11-17-beta-estradiol binding energy: -6.17 kcal/mol.

We searched the Herb database for 9 herbal medicines from BSHXP currently used to treat varicocele corresponding to the identified traditional Chinese medicine monomers (17-beta-estradiol and mannose-b) (**Figure 3A**). Molecular docking simulations showed that the binding relationship between target genes and herbal medicine molecules is stable. MEGF9-mannose-b binding energy: -1.74 kcal/mol, MLLT11-17-beta-estradiol binding energy: -6.17 kcal/mol (**Figures 3B, C**). Negative values usually mean that the drug molecule tends to bind to the target, indicating a mutual attraction between the target and the herb, potentially forming a stable protein-drug complex. Overall, 17-beta-estradiol and mannose-b, which interact with MEGF9 and MLLT11, are the current target proteins of varicocele herbal medicine, which suggests that BSHXP may act on 17-beta-estradiol and mannose-b to regulate MEGF9 and MLLT11 we have identified to achieve therapeutic purposes.

### Causal effects between MEGF9, MLLT11 and MI

**Figure 4:**
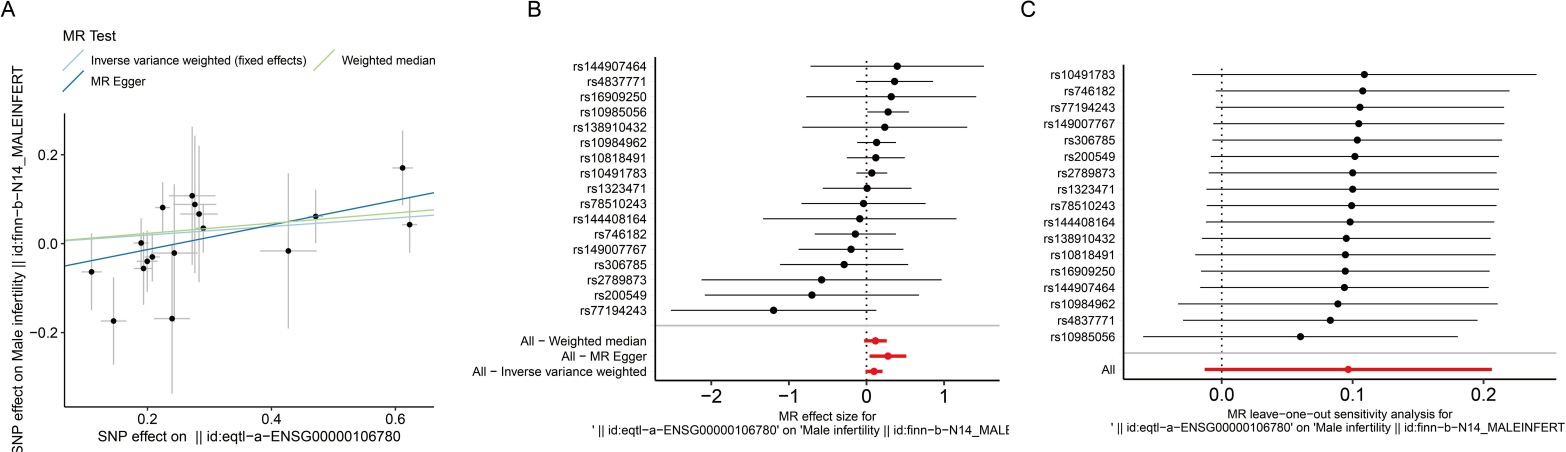
Effect of MEGF9 on MI. **(A)** Scatterplot showing the distribution of individual rate estimates for MEGF9 as a result of MI. Each scatterplot also contains trendlines derived from 3 different MR methods to indicate causality. **(B)** MR analysis forest plot of the association between MEGF9 and MI. The circles next to each SNP represent causal estimates for each IV, respectively, and the bottom three circles show multiple-instrument MR analysis using WM, Egger regression and IVW methods. Horizontal lines denote 95% CIs. **(C)** MR leave-one-out sensitivity analysis, used to estimate the causal effect of MEGF9 on MI, each black point represents an IVW, the red point represents the estimated value using all IVs, and the horizontal line represents the 95% CIs. MI, male infertility. MR, Mendelian randomization. IV, instrumental variables. CIs, confidence intervals. WM, Weighted median. IVW, Inverse variance weighted.

To assess the causal effect between MEGF9, MLLT11 and MI, we employed three-step two-sample MR analysis in this work. For the GWAS data that are significantly associated with the above disease phenotypes, after excluding LD for SNPs that caused bias and low quality, 15 and 13 SNPs were retained as IVs (*P* < 5×10^-8^), respectively. Our results found a significant causal relationship between MEGF9 and MI in the European population (MR Egger, OR: 1.639, 95% CI: 1.124-2.391, *P* = 0.024; WM, OR: 1.235, 95% CI: 1.003-1.521, *P* = 0.047) (**Supplementary Table S2**, **Figures 4A, B**).

To evaluate the MR hypothesis in the work, we selected SNPs with a genome-wide significance level of *P* < 5×10^-8^ to meet our first condition. Leave-one-out sensitivity analysis showed that deleting any SNPs did not significantly change the results, indicating the reliability of the results (**Figure 4C**). Cochran’s Q test was applied to assess the heterogeneity among the selected SNPs, and the results showed that neither MR Egger nor IVW analysis had statistically significant heterogeneity (*P* > 0.05). No evidence of directional pleiotropy was found as measured by MR-Egger regression (*P* for intercept > 0.05) (**Supplementary Table S2**). The above results verified our hypothesis that the SNPs used as IVs were significantly associated with MEGF9, and the causal estimate between MEGF9 and the risk of EI didn’t receive confounding factors.

**Figure 5:**
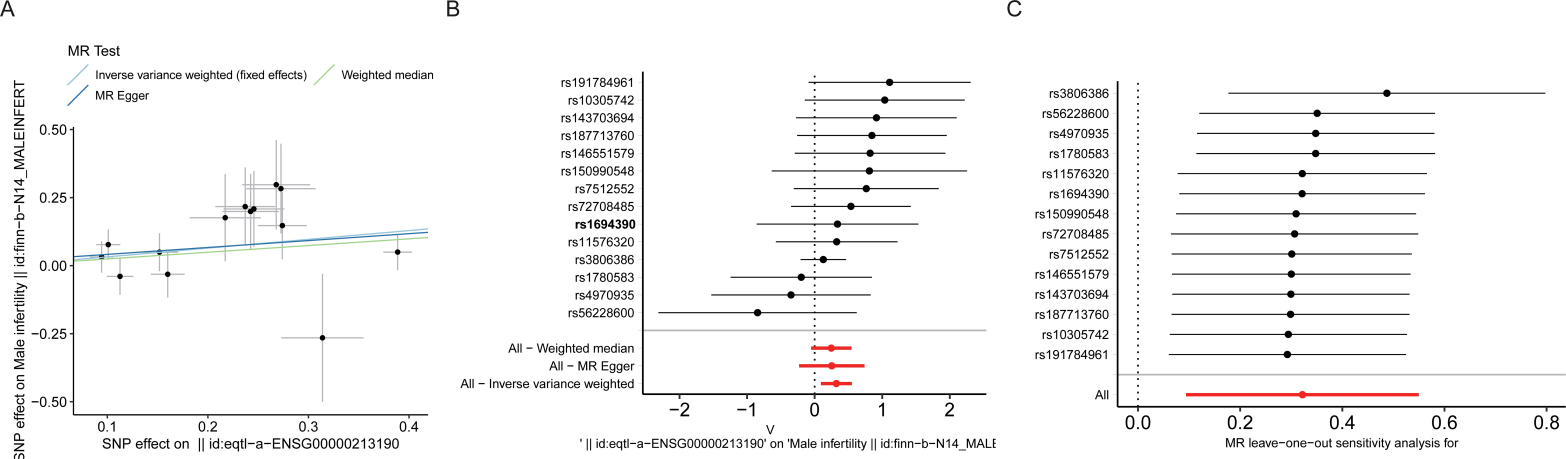
Effect of MLLT11 on MI. **(A)** Scatterplot showing the distribution of individual rate estimates for MLLT11 as a result of MI. Each scatterplot also contains trendlines derived from 3 different MR methods to indicate causality. **(B)** MR analysis forest plot of the association between MLLT11 and MI. The circles next to each SNP represent causal estimates for each IV, respectively, and the bottom three circles show multiple-instrument MR analysis using WM, Egger regression and IVW methods. Horizontal lines denote 95% CIs. **(C)** MR leave-one-out sensitivity analysis, used to estimate the causal effect of MLLT11 on MI, each black point represents an IVW, the red point represents the estimated value using all IVs, and the horizontal line represents the 95% CIs. MI, male infertility. MR, Mendelian randomization. IV, instrumental variables. CIs, confidence intervals. WM, weighted median. IVW, inverse variance weighted.

We also conducted MR analysis between MLLT11 and MI. The results showed that MLLT11 was significantly positively correlated with MI (IVW, OR: 1.35, 95% CI: 1.069-1.705, *P* = 0.012). Sensitivity analysis showed that the SNPs used as IVs were significantly associated with MLLT11, and the causal estimate between MEGF9 and the risk of EI didn’t receive confounding factors (**Figure 5**, **Supplementary Table S3**).

### Validation of MEGF9 and MLLT11 as potential targets for the treatment of varicocele

**Figure 6:**
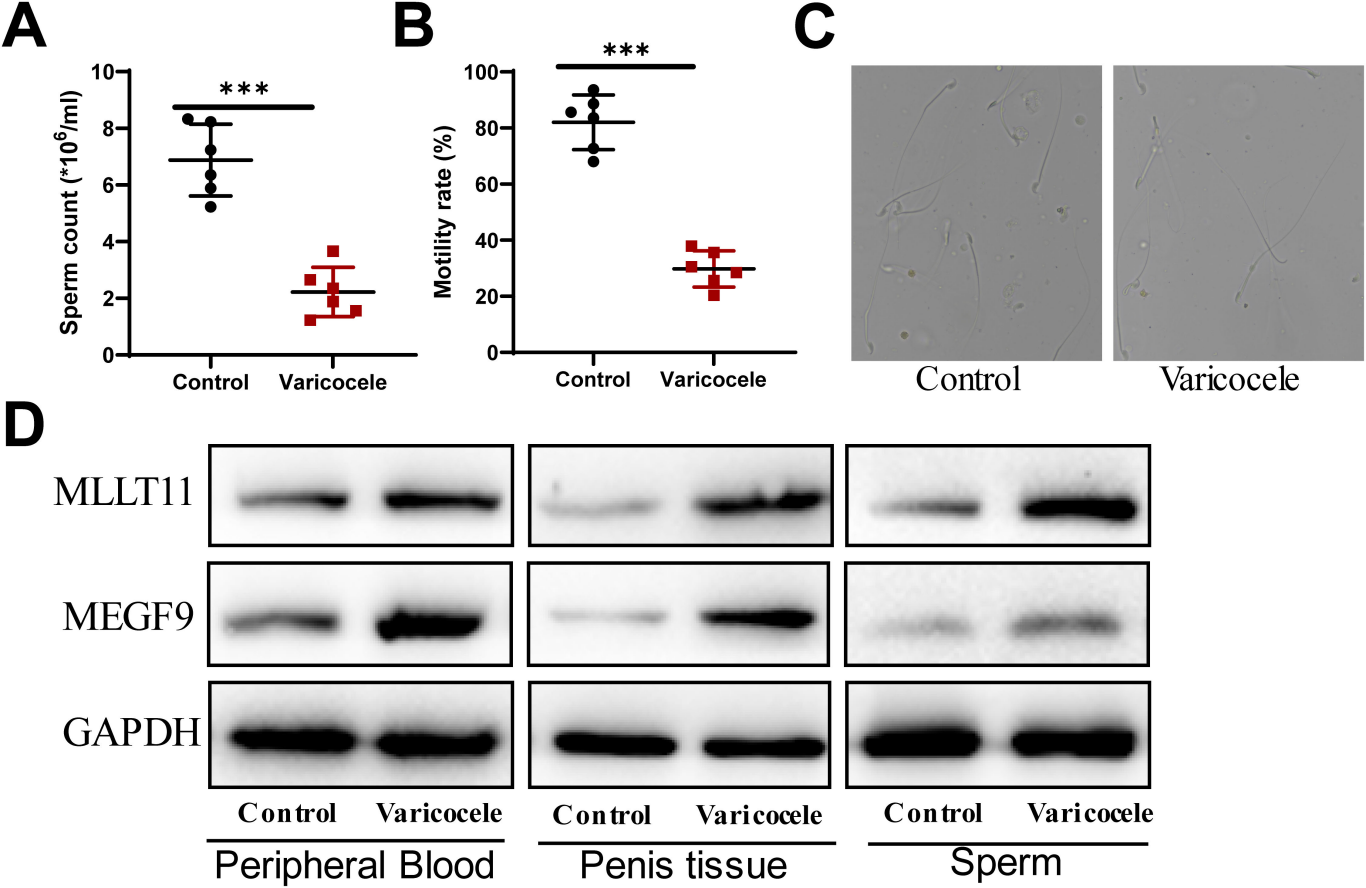
The expression of MEGF9 and MLLT11 is up-regulated in the peripheral blood, penile tissue, and sperm of rats in the model group. **(A)** Sperm counts were counted on a hemocytometer. **(B)** Observe sperm activity under a microscope and calculate the sperm activity percentage. **(C)** Observation of rat sperm morphology under an electron microscope. **(D)** The expression levels of MEGF9 and MLLT11 in the peripheral blood, penile tissue, and sperm of rats in the model group and control group were detected by WB. ***: P < 0.001.

Varicocele can lead to a significant decrease in fertility and is one of the main causes of male infertility (26). The detection of semen quality is the most direct indicator of fertility. The results of this experiment showed that the sperm number and sperm survival rate of rats in the model group were significantly lower than those in the control group (**Figures 6A, B**). Observed under the microscope, the outline of some sperm in the rats in the model group was not obvious, and the tail development was not completed (**Figure 6C**). WB results showed that compared with the control group, the expression of MEGF9 and MLLT11 was significantly up-regulated in the peripheral blood, penile tissue, and semen of rats in the model group (**Figure 6D**). This further validates the results from our above analysis.

**Figure 7:**
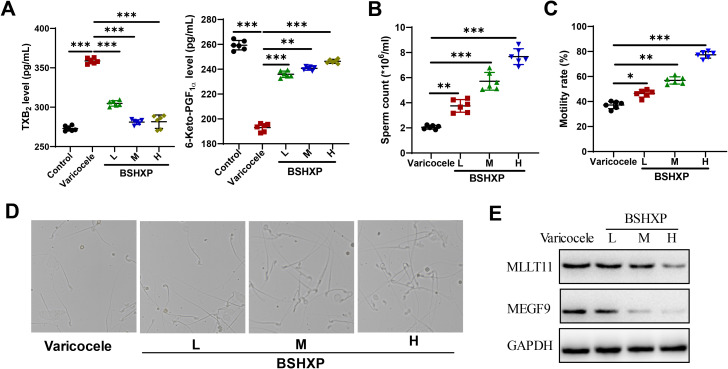
BSHXP has a good effect on promoting blood circulation and removing blood stasis. **(A)** ELISA detects TXB_2_ and 6-Keto-PGF_1_a levels in different groups. **(B, C)** Calculation of sperm count and motility after treatment with low, medium, and high doses of BSHXP. **(D)** Observation of sperm morphology after treatment with low, medium, and high doses of BSHXP. **(E)** The expression levels of MEGF9 and MLLT1 after BSHXP treatment were detected by WB. *: P < 0.05; **: P < 0.01; ***: P < 0.001.

Blood stasis is one of the basic pathogeneses of varicocele infertility. TXA2 and PGI2 are two important components of the prostaglandin family. Under normal physiological conditions, TXA2/PGI maintains dynamic balance, maintains vascular tone, and jointly participates in maintaining coronary artery tension and maintaining blood flow. Once imbalanced, it can lead to thrombosis and tissue ischemia (27). Since TXA2 and PGI2 are unstable and difficult to detect, TXB2 and 6-Keto-PGF1a are metabolites of TXA2 and PGI2 and are more stable in nature. By detecting the ratio of the two metabolites, the levels of TXA2 and PGI2 can be indirectly reflected. Experimental results show that TXB2 in plasma is significantly increased and PGF1a is significantly decreased in varicocele, indicating that the TXB2/PGF1a balance is imbalanced in varicocele. After treatment, the TXB2/PGF1a ratio increased (**Figure 7A**). In addition, sperm quality performed best in the BSHXP high-dose group (**Figures 7B–D**). We also found that the expression of MEGF9 and MLLT11 gradually decreased after low, medium, and high doses of BSHXP treatment (**Figure 7E**). This suggests that BSHXP has a good effect on promoting blood circulation and removing blood stasis, and may treat varicocele infertility by regulating MEGF9 and MLLT11 targets.

## Discussions

The benefits and harms of varicocele treatment in adult infertile men remain controversial (19). Varicocele can have deleterious effects on testicular function, leading to reduced semen quality, impaired sperm function, and compromised pregnancy outcomes in some men. Current evidence supports varicocele repair in men in infertile couples with clinical varicocele and affected semen parameters. The evidence does not support correction of subclinical varicocele. Bilateral varicocele repair should be performed only when the varicocele is palpable on both sides (26). Therefore, it is particularly critical to find effective and reliable drug treatments. Current research has confirmed that (28) BSHXP (Epimedium brevicornu Maxim, Rehmannia glutinosa, Hominis Placenta, Astragali Radix, Polygonatum sibiricum etc) is the basic prescription for treating this disease. It nourishes the kidneys to produce body fluids and activates blood circulation to remove blood stasis, thus improving the quality of reproductive essence. Data from recent molecular and genetic studies may provide new clues to understand the pathophysiology of the disease and to develop new diagnostic methods and treatment plans based on these findings.

MR analysis does not provide direct evidence of a causal relationship between varicocele and MI. However, we found significant causal effects between the target genes MEGF9 and MLLT11, which are abnormally upregulated in varicocele, and MI, providing indirect evidence for the possibility that varicocele causes MI. This suggests that MI may benefit from alternative treatments for varicocele. It has been reported that varicocele appears to cause many changes in the testicular microenvironment. These changes in temperature, reactive oxidative species, hemodynamics and antioxidant concentrations have been shown to have deleterious effects on sperm (29). Data show that the average temperature of the scrotum of normal men is 33.5°C, while the average temperature of men with varicocele is 35°C (30). Retrograde venography studies in men with varicocele have documented valvular insufficiency or insufficiency throughout the internal spermatic vein, with retrograde blood flow leading to varying degrees of increased hydrostatic pressure, prolonged stagnation time, and increased temperature within the testicles. The thermal reaction adversely affects the seminiferous epithelium, leading to the loss of spermatocytes and sperm cells (31). This is consistent with the conclusion of our MR analysis. Considering the importance of gene drug targets in successfully obtaining market approval, we used molecular docking of MEGF9 and MLLT11 with corresponding Traditional Chinese medicine monomers to treat varicocele to verify this finding. After establishing a pathological model of varicocele through renal vein constriction, it was found that varicocele led to a decrease in semen quality and blood stasis in rats. BSHXP treatment can significantly improve the semen quality of model rats, promote blood circulation, and improve their fertility. Among them, the high-dose group has the best effect. And we further verified that the expression of MEGF9 and MLLT11 was up-regulated in rats in the model group, and the expression gradually decreased after low, medium, and high doses of BSHXP treatment. Therefore, MEGF9 and MLLT11 may be two promising new drug targets for alleviating varicocele infertility. BSHXP has a good effect on promoting blood circulation and removing blood stasis, and may treat varicocele infertility by regulating MEGF9 and MLLT11 targets. This finding deserves further exploration.

Multiple EGF like domains 9 (MEGF9) is a novel transmembrane protein with multiple epidermal growth factor (EGF)-like repeats that is mainly expressed in the developing and adult nervous system (32). Current research shows that MEGF9 is related to various human diseases (33, 34). To our knowledge, the direct relationship between MEGF9 and male infertility or varicocele has not been fully studied. However, some studies have shown that MEGF9 may be related to some diseases related to the reproductive system and fertility. Specifically, MEGF9 has been found to be associated with polycystic ovary syndrome in some studies (35). The disease is a common and even systemic disorder that may lead to infertility in women. In addition, MEGF9 may mediate the adhesion of spermatogonia to supporting cells during sperm development (36). Local environmental changes caused by varicocele (such as increased temperature and oxidative stress in the testis) may lead to abnormal adhesion function mediated by MEGF9 (37), affecting sperm maturation. Varicocele may also lead to abnormal signal transduction pathways involving MEGF9 (32), thereby affecting sperm production. MLLT11 transcription factor 7 cofactor (MLLT11), also known as AF1Q, is a protein that acts as a transcription factor. MLLT11 usually attracts attention because of its involvement in chromosomal translocations found in certain types of leukemia, and is an oncogenic factor involved in metastasis of various types of cancer (38, 39). Similar to MEGF9, MLLT11 also regulates neuronal growth during development (40). To date, there are no literature reports on the association between MLLT11 and male infertility or varicocele. Therefore, a large number of prospective experiments are needed to verify. Reactive oxygen species (ROS) produced by oxidative stress have become an important pathogenic factor in the entire course of varicocele (41). MLLT11 may regulate cell survival in response to oxidative stress and may affect various stages of spermatogenesis by regulating the proliferation and differentiation of spermatogonia (42).

One limitation of this study is the limitation of the data set. We were unable to obtain relevant data on varicocele, so no MR analysis between varicocele and MI was performed. However, the results of this study showed that the target genes MEGF9 and MLLT11 were abnormally up-regulated in varicocele, and their expression decreased after BSHXP treatment. MR analysis showed that MEGF9 and MLLT11 are risk factors for MI, which indirectly proves the genetic causal relationship between varicocele and MI.

## Data Availability

The raw data supporting the conclusions of this article will be made available by the authors, without undue reservation.

## Supplementary material

Supplementary Figure 1 Intersection analysis of down-regulated genes in varicocele, genes negatively correlated with MI occurrence, and herbal targets.

Supplementary Table 1 311 genes positively correlated with the occurrence of MI.
