# Supplementary figures and images for: Mendelian randomization analysis and validation supports MEGF9 and MLLT11 as potential targets for the treatment of varicocele and male infertility
Source: Front Endocrinol (Lausanne). 2024 Sep 26;15:1416384. doi: 10.3389/fendo.2024.1416384 (PMC11464449; doi:10.3389/fendo.2024.1416384)

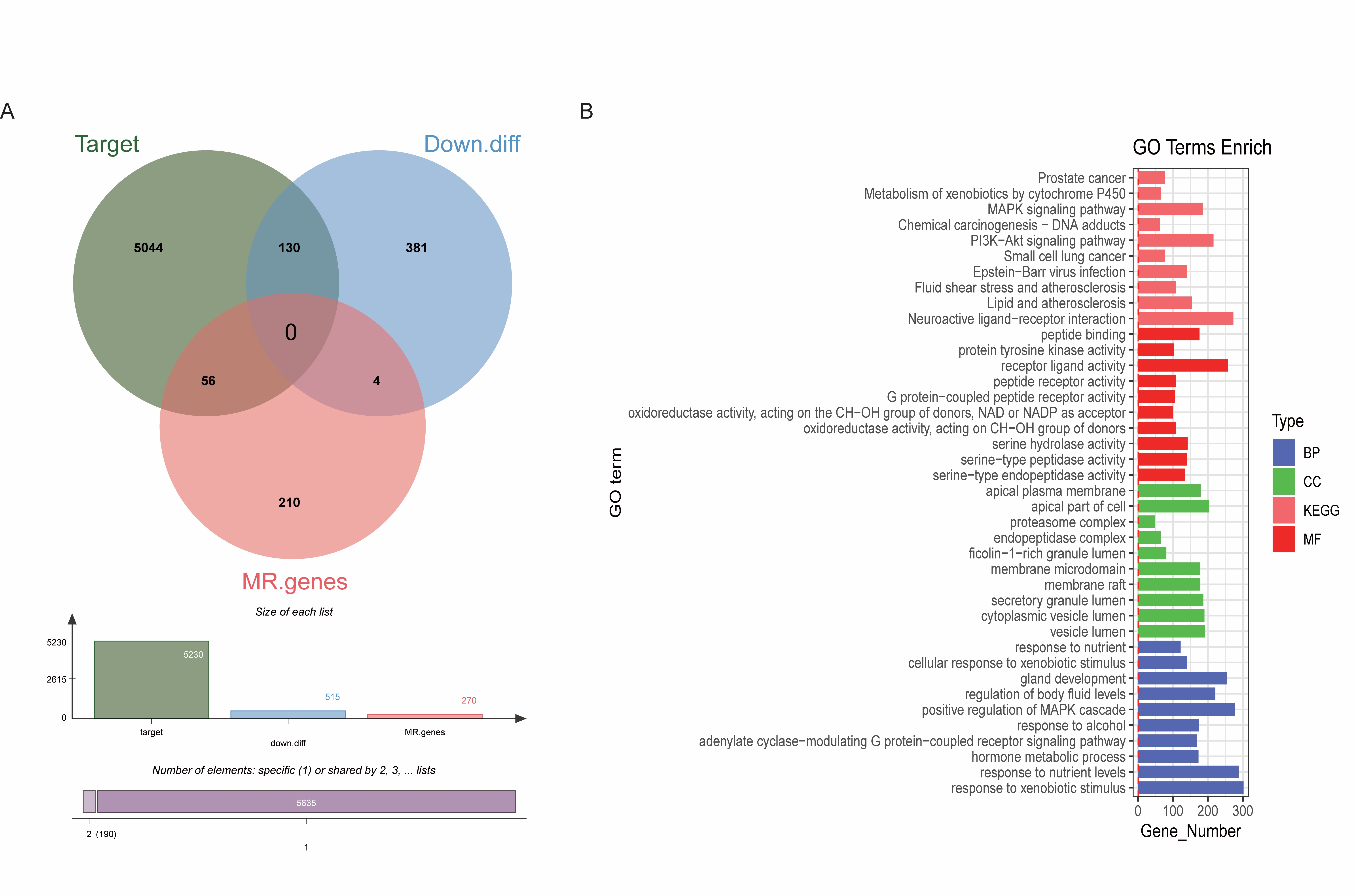

Supplement: Supplementary Figure 1 — Intersection analysis of down-regulated genes in varicocele, genes negatively correlated with MI occurrence, and herbal targets. [file Image1.jpeg]
