# Supplementary material for: Mendelian randomization analysis and validation supports MEGF9 and MLLT11 as potential targets for the treatment of varicocele and male infertility
Source: Front Endocrinol (Lausanne). 2024 Sep 26;15:1416384. doi: 10.3389/fendo.2024.1416384 (PMC11464449; doi:10.3389/fendo.2024.1416384)
Supplement: Supplementary file 3 [file Table2.docx]

**Table S2**. Significant causal relationship between MEGF9 and MI.

| **Exposure** | **Outcome** | **Methods** | **nsnp** | **β** | **se** | **pval** | **or** | **or_lci95** | **or_uci95** | **Q** | **Q_pval** | **egger_intercept** | **pval_intercept** |
| --- | --- | --- | --- | --- | --- | --- | --- | --- | --- | --- | --- | --- | --- |
| MEGF9 | MI | MR Egger | 15 | 0.277 | 0.121 | 0.024 | 1.639 | 1.124 | 2.391 | 9.061 | 0.874 | -0.069 | 0.113 |
| MEGF9 | MI | WM | 15 | 0.115 | 0.071 | 0.047 | 1.235 | 1.003 | 1.521 |  |  |  |  |
| MEGF9 | MI | IVW | 15 | 0.097 | 0.056 | 0.201 | 1.106 | 0.948 | 1.291 | 11.898 | 0.751 |  |  |
