# Supplementary material for: Mendelian randomization analysis and validation supports MEGF9 and MLLT11 as potential targets for the treatment of varicocele and male infertility
Source: Front Endocrinol (Lausanne). 2024 Sep 26;15:1416384. doi: 10.3389/fendo.2024.1416384 (PMC11464449; doi:10.3389/fendo.2024.1416384)
Supplement: Supplementary file 4 [file Table3.docx]

**Table S3**. Significant causal relationship between MLLT11 and MI.

| **Exposure** | **Outcome** | **Methods** | **nsnp** | **β** | **se** | **pval** | **or** | **or_lci95** | **or_uci95** | **Q** | **Q_pval** | **egger_intercept** | **pval_intercept** |
| --- | --- | --- | --- | --- | --- | --- | --- | --- | --- | --- | --- | --- | --- |
| MLLT11 | MI | MR Egger | 13 | 0.254 | 0.247 | 0.381 | 1.258 | 0.769 | 2.058 | 12.770 | 0.386 | 0.016 | 0.757 |
| MLLT11 | MI | WM | 13 | 0.246 | 0.071 | 0.155 | 1.259 | 0.921 | 1.722 |  |  |  |  |
| MLLT11 | MI | IVW | 13 | 0.322 | 0.056 | 0.116 | 1.35 | 1.069 | 1.705 | 12.876 | 0.457 |  |  |
